# Oncolytic Immunotherapy: Dying the Right Way is a Key to Eliciting Potent Antitumor Immunity

**DOI:** 10.3389/fonc.2014.00074

**Published:** 2014-04-10

**Authors:** Zong Sheng Guo, Zuqiang Liu, David L. Bartlett

**Affiliations:** ^1^Department of Surgery, University of Pittsburgh Cancer Institute, University of Pittsburgh School of Medicine, Pittsburgh, PA, USA

**Keywords:** immunogenic cancer cell death, DAMPs, PAMP, autophagy, tumor-associated antigen, cross-presentation, immune tolerance, antitumor immunity

## Abstract

Oncolytic viruses (OVs) are novel immunotherapeutic agents whose anticancer effects come from both oncolysis and elicited antitumor immunity. OVs induce mostly immunogenic cancer cell death (ICD), including immunogenic apoptosis, necrosis/necroptosis, pyroptosis, and autophagic cell death, leading to exposure of calreticulin and heat-shock proteins to the cell surface, and/or released ATP, high-mobility group box 1, uric acid, and other damage-associated molecular patterns as well as pathogen-associated molecular patterns as danger signals, along with tumor-associated antigens, to activate dendritic cells and elicit adaptive antitumor immunity. Dying the right way may greatly potentiate adaptive antitumor immunity. The mode of cancer cell death may be modulated by individual OVs and cancer cells as they often encode and express genes that inhibit/promote apoptosis, necroptosis, or autophagic cell death. We can genetically engineer OVs with death-pathway-modulating genes and thus skew the infected cancer cells toward certain death pathways for the enhanced immunogenicity. Strategies combining with some standard therapeutic regimens may also change the immunological consequence of cancer cell death. In this review, we discuss recent advances in our understanding of danger signals, modes of cancer cell death induced by OVs, the induced danger signals and functions in eliciting subsequent antitumor immunity. We also discuss potential combination strategies to target cells into specific modes of ICD and enhance cancer immunogenicity, including blockade of immune checkpoints, in order to break immune tolerance, improve antitumor immunity, and thus the overall therapeutic efficacy.

## Introduction

Oncolytic viruses (OVs) have been shown to be effective in treating cancer in preclinical models and promising clinical responses in human cancer patients (1–3). OV-mediated cancer therapeutic includes three major mechanisms. The first is the direct infection of cancer and endothelial cells in the tumor tissue leading to direct oncolysis of these cells. The second is necrotic/apoptotic death of uninfected cells induced by anti-angiogenesis and vasculature targeting of the OVs as shown in both animal models and human cancer patients (4–6). The last is the activated innate and adaptive tumor-specific immunity, which exert cytotoxicity to surviving cancer and stromal cells. A number of recent studies have demonstrated that the antitumor immunity has played an important role in the overall efficacy of oncolytic virotherapy, which has been shown to contribute to the efficacy of oncolytic virotherapy (7–14). In the case of oncolytic vesicular stomatitis virus (VSV), reovirus, and herpes simplex virus (HSV), the antitumor immune response is very critical to the overall efficacy of oncolytic virotherapy, sometimes even more important than that of direct oncolysis (7, 9, 11, 14).

Oncolytic viruses provide a number of potential advantages over conventional cancer therapies. First, OVs are tumor-selective antitumor agent, thus providing higher cancer specificity and better safety margin. Second, OV-mediated oncolysis not only leads to regression of tumor size, but this process provides key signals to dendritic cells (DCs) and other antigen presenting cells to initiate a potentially potent antitumor immune response. The immunogenic types of cell death induced by OVs provide danger signal (signal 0) and a natural repertoire of tumor-associated antigens (TAAs) to DCs, both required to trigger an adaptive immunity against cancer (15–17). The danger signals include damage-associated molecular pattern (DAMP) and pathogen-associated molecular pattern (PAMP) molecules derived from the OVs. Therefore, this process could provide a highly favorable immunological backdrop for the host to respond and generate potent adaptive antitumor immunity. However, just like other immunotherapeutic regimens for cancer, a number of challenges remain for OVs-mediated immunotherapy. One is that relative inefficiency of delivering OVs to tumor nodules, viral replication within tumor mass, and spread to distant metastases dampens its overall efficacy. Second, most TAAs are self-antigens and thus weakly immunogenic. As we will discuss below, OVs may enhance tumor immunogenicity in many cases. Yet, this low immunogenicity still is a problem due to the highly immunosuppressive tumor microenvironment (TME). Third, a highly immunosuppressive TME in late stages of cancer often suppresses the activities of tumor-infiltrated lymphocytes (TILs) generated either spontaneously or by an immunotherapeutic regimen (18).

In this review, we will discuss different modes of cell death induced by various OVs, their potential effects on the subsequent antitumor immunity. Then we discuss rationales and strategies of inducing ideal types of cancer cell death by either genetic modification on OVs or by combination with specific antitumor agents that lead to specific mode of immunogenic cancer cell death (ICD). Finally, we provide some perspective on future combination strategies to improve antitumor immunity for enhanced overall efficacy of virotherapy.

## OV: Tumor Selectivity and Relevance of Animal Model

Ideally, OVs selectively infect and replicate in cancer cells and cancer-associated endothelial cells, leading to direct oncolysis and subsequent antitumor activities without harming normal tissue (1–3). Some OVs display intrinsic tumor tropism (naturally occurring OVs), while others obtain their tumor selectivity through natural evolution or genetic engineering. The mechanisms underlying the tumor selectivity may include altered signaling pathways of ataxia telangiectasia mutated (ATM), epidermal growth factor receptor (EGFR), p53, PKR, Ras, RB/E2F/p16, Wnt, anti-apoptosis, or defects in cellular innate immune signaling pathways or hypoxia conditions in the TME (1, 3, 19, 20).

Viruses display strict viral tropism, specific for a cell type, tissue, or species. However, OVs often broaden their tropism to cancer cells from non-permissive species to various degrees. As an example, human adenovirus (Ad) does not infect normal murine cells, yet infect murine cancer cells even though the production of infectious virus progeny is often limited. A recent study may provide some answer to this phenomenon. McNeish et al. have found that murine cancer cells support viral gene transcription, mRNA processing, and genome replication of human Ad, but there is a profound failure of viral protein synthesis, especially late structural proteins with reduced loading of late mRNA onto ribosomes. Interestingly, *in trans* expression of the non-structural late protein L4-100K increases both viral mRNA loading on ribosomes and late protein synthesis, accompanied by reduced phosphorylation of eIF2α and improved anticancer efficacy (21). The key point is that some OVs display aberrant, non-productive infection in non-native hosts such as mouse cells, leading to mode of cancer cell death different from the mode of cell death in native host. As we will discuss extensively later, the mode of cancer cell death dictates to a significant degree the subsequent antitumor immunity. As a consequence, the OV-elicited antitumor immunity in tumor models of syngeneic animals might not be relevant to the situation in human cancer patients. This is an often overlooked issue when tumor models in animals are chosen along with OVs as therapeutic models for human cancer.

## Signal 0: DAMPs and PAMPs

### PAMPs: Signal 0s from pathogens

In the late 1980s, Charles Janeway proposed that the immune system protects the host against infectious pathogens by presenting the molecules as signal 0s, which is what now called PAMPs, to the antigen presenting cells (22, 23). PAMPs consist of essential components of microorganisms that direct the targeted host cells, key components in the innate immune arm, to distinguish “self” from “non-self,” and promote signals associated with innate immunity (24). Major PAMPs are nucleic acids (DNA, double-stranded RNA, single-stranded RNA, and 5′-triphosphate RNA), proteins (lipoproteins and glycoproteins), as well as other components of the cell surface and membrane (17, 25). Interestingly, defective viral genomes arising *in vivo* are a critical danger signal for triggering antiviral immunity in the lung (26).

This concept of PAMPs has been strongly supported by the discovery of several classes of pattern-recognition receptors (PRRs). These PRRs include the toll-like receptors (TLRs), retinoic acid-inducible gene-1 (RIG-1)-like receptors (RLRs), nucleotide oligodimerization domain (NOD)-like receptors (NLRs), AIM2-like receptors, and the receptor for advanced glycation end products (RAGE) (17, 27). It is now well accepted that both DAMPs and PAMPs stimulate the innate immune system through PRRs. DCs express a wide repertoire of these PRRs. The binding of PAMP to its receptors on the APC activates the DCs (28, 29).

### DAMPs: Signal 0s from host

Matzinger proposed what is known now as the “danger theory” in 1994 (30). In the theory, it proposed that the immune system can distinct self from non-self and dangerous from innocuous signals. In this model, APCs are activated by both PAMPs and DAMPs from distressed or damaged tissues or microbes. The theory has been well accepted in recent years, as we have learned more and more about how dying cells alert immune system to danger (31). Over the years, a number of endogenous danger signals have been discovered. For examples, it was shown that uric acid functions as a principal endogenous danger signal, which is released from injured cells (32).

Damage-associated molecular patterns are molecules derived from normal cells that can initiate and perpetuate immunity in response to cell stress/tissue damage in the absence of pathogenic infection. DAMPs vary greatly depending on the type of cell and injured tissue. They can be proteins, DNA, RNA, or metabolic products. Protein DAMPs include intracellular proteins, such as high-mobility group box 1 (HMGB1), heat-shock proteins (HSPs), and proteins in the intracellular matrix that are generated following injury, such as hyaluronan fragments (33). HMGB1 is one prototypic DAMP (34, 35). The protein DAMPs can be localized within the nucleus, cytoplasm, cell membrane, and in exosomes, the extracellular matrix, or as plasma components (17). Other types of DAMPs may include DNA, ATP, uric acid, and heparin sulfate. It is interesting to note that mitochondria are a rich and unique source of DAMPs, including formyl peptides, the mitochondrial DNA (mtDNA)-binding proteins, transcription factor TFAM, and mtDNA itself (36). Following interactions between DAMPs and PRRs on the target cells, the intracellular signaling cascades triggered by the interactions between DAMPs and PRRs lead to activation of genes encoding inflammatory mediators, which coordinate the elimination of pathogens, damaged, or infected cells (27). In cancer, chronic inflammation and release of DAMPs promotes cancer, while acute inflammation of release/presentation of DAMPs may induce potent antitumor immunity and helps in cancer therapy (35, 37). Based on the work in chemotherapy and radiation therapy, the concept of ICD of cancer cells has been established about 10 years ago (37, 38). As we will discuss below, this concept leads to development of novel strategies for cancer therapeutics.

## OVs Induce Mostly Multimodality ICD and Release/Present Danger Signal Molecules

**Figure 1:**
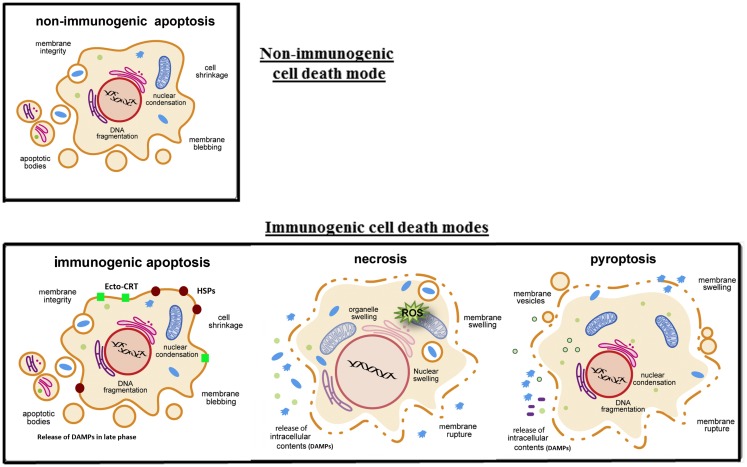
**Four key modes of cancer cell death and their immunogenicity**. In classic apoptosis, the retention of plasma membrane integrity and the formation of apoptotic bodies render it an immunologically silent death mode, or non-immunogenic cell death. However, recent studies have shown that cancer cells treated with certain cytotoxic agents (some chemotherapeutic agents and oncolytic viruses) lead to the cell surface exposure of calreticulin (ecto-CRT) and heat-shock proteins (HSPs) prior to apoptosis, and other DAMPs released in the later phase of apoptosis, danger signals to DCs. This is immunogenic apoptosis. Cancer cells dying by necrosis/necroptosis or pyroptosis secrete pro-inflammatory cytokines and release their cytoplasmic content, including DAMPs (ATP, HMGB1, and uric acid, etc.), into the extracellular space. Some DAMPs (such as HMGB1) can be secreted through non-classical pathways (25). These later modes of cancer cell death are ICD. Drawings are modified and reprinted from Lamkanfi and Dixit (47), copyright 2010, with permission from Elsevier.

Investigators have long been interested in what defines the immunogenicity of cancer cells and how we can enhance the immunogenicity for the purpose of immunotherapy. Pioneering work by Lindenmann and Klein almost half a century ago demonstrated that viral oncolysis of cancer cells by influenza virus increases immunogenicity of tumor cell antigens (39). However, it was not clear how this immunogenicity was enhanced at the time. Over a decade ago, it was found that tumor immunogenicity is enhanced by cell death via induced expression of HSPs (40). A few years ago, investigators working on chemotherapy and radiation for cancer therapy have led to this new concept as they classify the types of cancer cell death by the immunological consequence, into “immunogenic cancer cell death” (ICD) and “non-immunogenic cancer cell death” (NICD) (41–43). The original concept of ICD includes only “immunogenic apoptosis.” We and others have recently proposed that ICD includes not only immunogenic apoptosis, but also necroptosis, necrosis, autophagic cell death, and pyroptosis of cancer cells (Figure 1) (44, 45). Basically, cancer cells dying via ICD have the following common features as summarized by Tesniere, Zitvogel, Kroemer, and their colleagues (46). They stated that, “some characteristics of the plasma membrane, acquired at pre-apoptotic stage, can alarm immune effectors to recognize and then attack these pre-apoptotic tumor cells. The signals that mediate the immunogenicity of tumor cells involve elements of the DNA damage response, elements of the endoplasmic reticulum stress response, as well as elements of the apoptotic response” (46). For cells undergoing pre-apoptotic phase, they may express “danger” and “eat-me” signals on the cell surface (calreticulin and HSPs) or can secrete/release immunostimulatory factors (cytokines, ATP, and HMGB1) to stimulate innate immune effectors (46). For other types of ICD, extracellular ATP, HMGB1, uric acid, other DAMPs, and PAMPs released in the mid or late phases functions as potent danger signals, thus making it highly immunogenic.

Oncolytic viruses kill cancer and associated endothelial cell through a variety of types of cell death as classically defined by the morphological and ultrastructural changes of dying cells. These include apoptosis, necrosis, necroptosis, pyroptosis, and autophagic cell death, often with one as the predominant form of death for a particular OV. By the new definition, cancer cell death induced by OVs is mostly immunogenic (Table 1). Probably all oncolytic Ads induced autophagic cell death in cancer cells (48–51). Coxsackievirus B3 (CVB3) induces immunogenic apoptosis in human non-small cell lung cancer cells (52). Measles virus (MV) causes ICD in human melanoma cells, because inflammatory cytokines and HMGB1 are released, and DCs are activated by MV-infected cancer cells (53). HMGB1 release often happens in late stage of apoptosis, during autophagy process and in necrotic cells infected with OVs. We first reported in 2005 that human cancer cells infected by an oncolytic poxvirus, led to necrotic/apoptotic death pathways and release of HMGB1 (54). Later studies have confirmed and extended the findings of HMGB1 release in cancer cells infected with Ads (12), CVB3 (52), an MV (53), vaccinia viruses (VVs) (55–57), HSV (14, 58), and parvovirus H-1 (H-1PV) (59). Extracellular ATP is another potent danger signal released from OV-infected cancer cells (12, 52, 56, 60). The third danger signal molecule released from OV-infected cells is uric acid (61). Some OVs may induce cell death partly through pyroptosis, a caspase-1 dependent inflammatory form of cell death (62). Both necrotic cells and pyroptotic cells release ATP more efficiently than apoptotic cells do. Pyroptotic cells, just like apoptotic cells, actively induce phagocytosis by macrophages using “eat-me” and “find-me” signals (63). Cytolytic immune cells, elicited by OVs or other agents, kill additional cancer cells leading to release of DAMPs such as HMGB1 (64). In summary, most OVs induce ICD of cancer cells and present/release a number of potent danger signals, and TAAs to DCs to trigger adaptive immune response (Table 1).

**Table 1:** **Oncolytic viruses lead to specific mode of immunogenic cell death and exposure/release of DAMPs/PAMPs**.

OV	DAMP/PAMP	Receptor	Type of cell death	Immunological functions	Reference
Ad5/3-D24-GM-CSF; CVB3; vvDD	ATP	P2Y2 and P2X7	Necrosis, autophagic cell death, and immunogenic apoptosis	Function as a “find-me” signal, and cause NLRP3-inflammasome-based IL-1β production	(52, 56, 60)
Ad5/3-D24-GM-CSF; CVB3	Ecto-CRT (calreticulin)	CD91	Immunogenic apoptosis (either pre-apoptotic, early or mid apoptotic surface exposure) or secondary necrosis	Function as an “eat-me” signal and it is a potent mediator of tumor immunogenicity crucial for elicidation of antitumor immunity	(52, 60)
Parvovirus H-1 (H-1PV)	HSPs: (HSP90, HSP70, Hsp72)	CD91, TLR2, TLR4, SREC1, and FEEL1	Immunogenic apoptosis (surface exposure) or necrosis (passively released)	Surfaced-exposed HSP90 can mediate adaptive antitumor immunity, while secreted HSP90 can inhibit TGF-β1 activation; Leads to TAA-specific antitumor immunity	(65–67)
? (Not identified)	Histones	TLR9	Apoptosis (cell surface exposure) or accidental necrosis (passively released)	Released histones can cause initiation of TLR9-MyD88-mediated inflammation	(68)
Many OVs: Ad; HSV; MV; VV; H-1PV	HMGB1	TLR2, TLR4, RAGE, and TIM3	Immunogenic apoptosis; necrosis; autophagic cell death	Activate macrophages and DCs; recruit neutrophils; promote *in vivo* the production of IFN-γ, TNF-α, IL-6, IL-12, and antigen-specific activation of CD8^+^ T cells	(53, 54, 56, 57, 59, 60)
MV-eGFP	IL-6	IL-6R and GP130	Necroptosis	A cell type-specific endokine DAMP with potent pro-inflammatory activity	(53)
Telomelysin (Ad)	Uric acid	P2Y6	Autophagic cell death	Stimulate the production of inflammatory cytokines such as IL-1, TNF-α, and IL-6 and chemotactic factors for neutrophils such as IL-8/CXCL8 and S100A8/A9	(61, 69)
Newcastle disease virus (NDV)	dsRNA and other PAMPs	TLR3; and by the cytoplasmic receptors MDA-5 and RIG-I	Immunogenic Apoptosis; autophagy	(1) Upregulation of HLA antigens and ICAM-1; (2) induction of type I IFNs and chemokines (CCL5 and CXCL10); (3) activate DCs and T effector cells but also to block Treg cells; (4) local therapy with oncolytic NDV induces inflammatory immune infiltrates in distant tumors, making them susceptible to systemic therapy	(70–74)
Reovirus	The virus itself (PAMP)	Dendritic cells (DCs)	(Cancer cell independent mechanism)	Induce DC maturation and stimulate the production of the pro-inflammatory cytokines IFN-α, TNF-α, IL-12p70, and IL-6. Reovirus directly activates human DC and that reovirus-activated DCs stimulate innate killing by not only NK cells, but also T cells	(75)

Cancer cell death induced by some OVs has not been examined for their direct features of ICD. However, other properties suggest that cancer cells infected by the OV are immunogenic, or the viruses themselves are highly immunogenic. Newcastle disease virus (NDV) is a well-studied virus for its virology and immunostimulatory properties (76). NDV induces cancer cells into apoptosis (70), with autophagy taking place during the process (71). Human cancer cells infected by NDV show upregulation of HLA class I and II antigens, and costimulatory molecule ICAM-1, as well as induction of IFNs, chemokines (IP10 and RANTES) before apoptosis (72). Moreover, the inflammatory conditions and type I IFNs inhibit Treg cells (73). With these potent immunostimulatory properties, local administration of oncolytic NDV overcomes systemic tumor resistance to immunotherapy by blockade of immune checkpoints (74). Another RNA virus, reovirus, also induces cancer cells into apoptosis (77, 78), with autophagy taking place in the process (79–81). Melanoma cells infected with reovirus release a range of inflammatory cytokines and chemokines while IL-10 secretion is abrogated (82). These molecules may provide a useful danger signal to reverse the immunologically suppressive environment of this tumor. Even more interestingly, reovirus can also interact with DCs directly and matured DCs activate NK and T cells (75) (Table 1). Those activated NK and T cells exert innate killing of cancer cells. This innate effector mechanism may complement the virus’s direct cytotoxicity and thus induced adaptive antitumor immunity, potentially enhancing the efficacy of reovirus as a therapeutic agent (75).

## OV-Induced Autophagy in Cancer Cells Promotes Cross-Presentation of TAAs and Elicits Stronger Antitumor Immunity

Autophagy mediates sequestration, degradation, and recycling of cellular organelles and proteins, and intracellular pathogens. It is not too surprising that autophagy plays roles in both innate and adaptive immunity (17, 83). A number of OVs, such as Ad (48–51), encephalomyocarditis virus (84), HSV (62, 85, 86), influenza virus (87), NDV (71), reovirus (79–81), and VSV (84), induce autophagy in infected cancer cells. Evidence shows that autophagy may enhance tumor immunogenicity. One mechanism is that autophagic cells selectively release DAMPs such as ATP (88, 89), HMGB1 (90), and uric acid (61). The other mechanism is that autophagy promotes antigen cross-presentation from cancer cells by DCs to naïve T cells. It stimulates antigen processing for both MHC class II (91), and MHC class I pathways. These have been demonstrated for endogenous viral antigens during HSV-1 infection (85), and for cross-presentation of TAAs from uninfected cancer cells (92), and influenza A virus-infected tumor cells (93). In other words, autophagy within the antigen donor cells facilitates antigen cross-priming to generate TAA-specific or virus-specific CD8^+^ T cells (92–95). This property has been explored for cancer vaccines (96), and for enhanced OV-mediated antitumor effects in the future (97).

## Viruses Often Encode Specific Genes to Modulate Apoptosis, Autophagy, Necroptosis, and Possibly Other Death Pathways

Successful viral replication requires the efficient production and spread of progeny virus, which can be achieved through efficient evasion of host defense mechanisms that limit replication by killing infected cells. Viruses have thus evolved to encode genes whose products function to block or delay certain cell death pathways until sufficient progeny have been produced (47). These gene-encode products target a variety of strategic points in apoptosis, necroptosis, autophagy, or other death pathways. Table 2 lists some examples of genes encoded by viruses especially OVs that can intervene apoptosis, autophagy, or necroptosis. The presence of these types of viral genes may skew the mode of infected cancer cells from one to another cell death pathway(s). OVs can be engineered genetically with deletion or insertion of such genes so that a desired mode of ICD would happen in the virus-infected cancer cells.

**Table 2:** **Examples of viruses and viral genes modulating apoptosis, autophagy, and necroptosis**.

Virus	Gene	Type of action	Mechanism of action	Reference
Ad	E1A	AS	Associate with the pRb/p300 family and induce p53-dependent apoptosis	(98)
	E1B-19K	AI	Sequester pro-apoptotic Bcl-2-like proteins and p53; inhibit apoptosis triggered by numerous stimuli	(99–101)
	E1B-55K	AI	Bind to p53 and functionally inactivates it	(102)
	E3-6.7	AI	Complexes with 10.4 and 14.5 resulting in downregulation of TRAIL receptors	(103)
HSV	ICP34.5	ATI	Inhibit PKR signaling and directly bind to beclin-1	(104)
	ICP34.5	AI	IFN-mediated pathway; decrease elF-2α phosphorylation by PKR	(105–107)
	Us3	AI	Ser/Thr kinase that prevents virus-induced apoptosis	(108)
	Us5	AI	Cooperates with Us3	(108)
VV	SPI-1		Serpin, inhibit cell-cell fusion	(109)
	SPI-2	AI	Serpin, direct inhibitor of caspases	(110)
	F1L	AI	Interact with the pro-apoptotic protein Bak and inhibit Bak activation	(111)
	N1L	AI	Inhibit multiple pro-apoptotic Bcl-2-like proteins	(112)
MYXV	M11L	AI	Prevent the mitochondria from undergoing a permeability transition; inhibit apoptotic response of macrophages and monocytes	(113, 114)
MCMV	vIRA	NI	Target RIP1, RIP3, TRIF, and DAI; inhibit RIP3-dependent necrosis	(115)
Influenza virus	M2	ATI	Block autophagosome fusion with lysosomes	(116)
	NS1	AI/ATS	Inhibit apoptosis and upregulate autophagy	(117)
Measles virus	H	AS	Induce apoptosis of HeLa cells via both extrinsic and intrinsic pathways	(118)
	Virion	ATS	Binding of virus to CD46 on cell surface induces autophagy	(119)
NDV	V	AI	Inhibit IFN response and apoptosis	(120)

*AI, apoptosis inhibitor; AS, apoptosis stimulator; NI, necroptosis inhibitor; ATI, autophagy inhibitor; ATS, autophagy stimulator*.

## Cancer Cells Often Show Defects in Certain Cell Death Pathways

Every cell in a multicellular organism has the potential to die by apoptosis. However, cancer cells often have faulty apoptotic signaling pathways evolved during carcinogenesis. This property derives from the overexpression of anti-apoptotic genes, deficiency of pro-apoptotic genes, or both (121). These defects not only increase tumor mass, but also render the cancer resistant to therapy.

Evidence has also been accumulating that necroptosis can be impaired in cancer cells. Chronic lymphocytic leukemia cells have defects in signaling pathways involved in necroptosis regulation such as RIP3 and the deubiquitination cylindromatosis (CYLD), an enzyme directly regulating RIP1 ubiquitination (122). Skin cancer cells contain an inactivating CYLD mutation (123). Despite the fact some cancers are resistant to necroptosis due to genetic and epigenetic defects, necroptosis undoubtedly represents an important death pathway induced by many anticancer regimens, particularly important to those cancer resistant to apoptosis. In this case, investigators have found that some compounds can circumvent cancer drug resistance by induction of a necroptotic death (124).

The fact that cancer cells resist certain death pathways will dictate to a degree which types of drugs (including OVs) to be used in therapeutic regimens. As we stated before, a number of OVs, such as VVs, often induces cancer cells into necroptotic cell death (54, 56, 57), while other viruses such as oncolytic Ad often induce cancer cells into autophagic cell death. Appropriate OVs can be picked depending on the sensitivity of the cancer to certain death pathways, and the immunogenic consequence if it is combined for immunotherapy.

## Strategies to Modulate the Mode of Cancer Cell Death for Enhanced Immunogenicity

We know now that immunogenic apoptosis, necrosis/necroptosis, and autophagic cell death are desired modes of cancer cell death because they are ICD. Is immunogenic apoptosis (the original form of ICD) better than other forms of ICD in the induction of antitumor immunity? We do not know for sure. This question needs to be addressed in the future. What we do know now is that there are strategies that can enhance the ICD and subsequent antitumor immunity. They can be classified into, genetic modification of OV vectors, combination with ICD inducers, and combination with specific immunostimulatory regimens.

### Genetic engineering of viral vectors

Cancer cells have usually accumulated a number of genetic mutations and epigenetic modifications that enable them to resist apoptosis. Based on this property, a number of OVs are built for high tumor selectivity by deleting viral genes encoding anti-apoptotic genes (see Table 2). These viruses can replicate in cancer cells but lead to rapid apoptosis in normal cells. For examples, the γ34.5 gene has been deleted in many oncolytic HSVs, including the T-VEC that is going through a successful phase III clinical trial (125). The adenoviral protein E1B-19K is a Bcl-2 homolog that blocks apoptosis induction via the intrinsic and extrinsic pathways, specifically including tumor necrosis factor (TNF)-mediated cell death. Liu et al. have demonstrated that an E1B-19K gene deletion mutant had TNF-enhanced cancer selectivity due to genetic blocks in apoptosis pathways in cancer cells (126). Similarly, a tumor-selective oncolytic vaccinia virus was constructed by deleting two serpin genes, SPI-1 and SPI-2 (54). Due to the deletion of viral anti-apoptosis genes, these mutant OVs display more potent oncolysis through apoptosis pathways when combined with appropriate apoptosis-inducing agents.

We believe that by arming OVs with necrosis and autophagy-promoting genes, it is possible that the desired cell death pathway can be activated in cancer cells when infected with such OVs, leading to more ICD. More future studies with this strategy are warranted.

### Combination with ICD inducer or autophagy inducer

In theory, OV in combination with an ICD inducer would provide more potent danger signals to DCs and potentially elicit stronger antitumor immunity. Workenhe et al. demonstrated in a recent study that such a strategy worked well indeed (127). HSV-1 ICP0 null oncolytic vectors possess antitumor activity, but the virus alone is insufficient to break immune tolerance. Thus, the authors hypothesized that combination therapy with an ICD-inducing chemotherapeutic drug might get the job done. Indeed, the combination of HSV-1 ICP0 null oncolytic virus with mitoxantrone, which induces ICD, provided significant survival benefit to the Balb/C mice bearing Her2/neu TUBO-derived mammary tumors. Increased infiltration of neutrophils and tumor antigen-specific CD8^+^ T cells into tumor tissues provide the protection, as depletion studies verified that CD8-, CD4-, and Ly6G-expressing cells are essential for the enhanced efficacy. Importantly, the combination therapy broke immune tolerance. In conclusion, this study suggests that such a combination can enhance the tumor immunogenicity, breaking immunologic tolerance established toward the tumor antigens, thus a promising novel strategy for cancer therapy (127).

As we stated earlier, the autophagy in antigen donor cells (cancer cells) promotes the cross-presentation of antigens from DCs to T cells. The autophagy could be induced by some OVs, or its inducer could be provided *in trans*. This strategy works in combination with oncolytic adenoviruses that induce autophagy by themselves (60, 128). However, it may not work with an oncolytic vaccinia virus that does not induce autophagy by itself (our unpublished data).

### Armed virus and combination strategies for breaking immune tolerance and enhancing antitumor immunity

In order to further enhance the antitumor immunity, OVs have been armed with TAAs, cytokines (e.g., GM-CSF), chemokines (such as CCL5), or other innovative and artificial genes. We have recently reviewed the promising strategies of OVs in combination with other immunotherapeutic regimens (44). As we mentioned, two OVs in the most advanced stages of clinical trials, T-VEC, and Pexa-Vec, are HSV and VV armed with GM-CSF (125, 129). An oncolytic VV expressing the 4-1BBL T cell costimulatory molecule (rV-4-1BBL) showed modest tumor regression in the poorly immunogenic B16 murine melanoma model. However, rV-4-1BBL injection with lymphodepletion promoted viral persistence by reducing antiviral antibody titers, and promoted MHC class I expression, and rescued effector-memory CD8^+^ T cells. This significantly improved the therapeutic effectiveness of the oncolytic virus (130). Similarly, an unarmed oncolytic virus combined with anti-4-1BB agonist antibody elicits strong antitumor immunity against established cancer (56). We have also shown that the chemokine CCL5-expressing oncolytic VV in combination with a cancer vaccine or activated T cells resulted in better therapeutic effect in a MC38 colon cancer model (131). Recently, our collaborators have made an oncolytic VV encoding a secretory bispecific T cell engager consisting of two single-chain variable fragments specific for CD3 and the tumor cell surface antigen EphA2 [EphA2-T cell engager-armed VV (EphA2-TEA-VV)] (132). This virus retains its normal oncolytic potency and the secreted molecule also activates T cells. The virus plus T cells had potent antitumor activity in a lung cancer xenograft model. Thus, arming oncolytic VVs with T cell engagers may represent a promising approach to improve oncolytic virotherapy. In the context of OV-mediated cancer immunotherapy, it is interesting to observe the dual effects of antiviral immunity on cancer therapy. On one hand, the antiviral immunity may attenuate the replication of an OV and thus diminish the effect of direct oncolysis; on the other hand, antiviral immunity plays a key role for the therapeutic success of oncolytic virotherapy in some cases (11, 133).

The tumor-associated immune tolerance is a big obstacle in cancer immunotherapy. Some armed OVs (such as a GM-CSF-armed oncolytic Ad) can break immune tolerance and generated antitumor immunity in at least some human cancer patients (134). In other cases, an OV alone is not enough to break the immune tolerance in highly immunosuppressive TME (127). In these cases, a combination with an ICD-inducing chemotherapeutic drug may break the immune tolerance (127). Alternatively, an OV can be combined with an immune checkpoint inhibitor to achieve the same effect. During the preparation of this review, a study has just been published on such a strategy with oncolytic NDV and systemic CTLA-4 blockade. This combination led to rejection of pre-established distant tumors and protection from tumor rechallenge in poorly immunogenic tumor models (74). It showcases the promise of such a combination strategy.

## Conclusion and Perspectives

The TME in the advanced stage of disease is highly immunosuppressive (18). This immunological property is a double-edged sword for OV-mediated cancer therapy: good for viral replication but bad for the antitumor immunity. The evidence is accumulating that OVs not only kill infected cancer cells and associated endothelial cells by direct and indirect oncolysis, but also release/present danger signals to DCs and other professional APCs to elicit both antiviral and antitumor immunity. It has been demonstrated for a number of OVs, that the virus-elicited antitumor immunity plays a critical role in the overall efficacy of oncolytic virotherapy. As we and other colleagues have realized, ICD is important to elicit antitumor immunity (44, 45, 135).

In order to improve the potency of antitumor immunity, one key step is the initial presentation of danger signal (signal 0) and cross-presentation of TAAs (signal 1). Recent studies demonstrated that ICD of cancer cells leads to potent danger signals, and autophagy in antigen donor cells, in this case cancer cells and associated endothelial cells, enhance the cross-presentation of TAAs to naïve T cells by DCs. Genetic engineering and combination strategies can skew the cancer cell death into modes of ICD and autophagy, leading to potent and sustained antitumor immunity and thus enhancing the efficacy of oncolytic immunotherapy. Which mode of ICD in the context of OVs is the most potent way to elicit antitumor immunity needs careful investigation in the near future. It is also important to keep in mind that oncolytic viruses modulate cancer immunogenicity through multiple mechanisms (136). Other than the induced danger signals, they are out of the scope of this review article and thus have not been discussed. Finally, we and others believe that it is important to further test the idea that combination of OV with blockade of immune checkpoints for potent and sustained antitumor immunity would enhance this novel form of immunotherapy for cancer. We look forward to more exciting development of both preclinical and clinical studies with OVs as tools for cancer immunotherapy.

## Author Contributions

Zong Sheng Guo collected and read relevant papers; designed and drafted the manuscript. David L. Bartlett and Zuqiang Liu have made suggestions to the manuscript. All authors have read and approved the final manuscript.

## Conflict of Interest Statement

David L. Bartlett is a scientific advisor for and has financial interest with Jennerex Biotherapeutics, a biopharmaceutical company developing oncolytic virotherapy. The other authors declare no conflict of interest.

## References

[1] Russell SJ Peng KW Bell JC Oncolytic virotherapy. Nat Biotechnol (2012) 30:658–70 10.1038/nbt.2287 22781695 PMC3888062

[2] Patel MR Kratzke RA Oncolytic virus therapy for cancer: the first wave of translational clinical trials. Transl Res (2013) 161:355–64 10.1016/j.trsl.2012.12.010 23313629

[3] Guo ZS Thorne SH Bartlett DL Oncolytic virotherapy: molecular targets in tumor-selective replication and carrier cell-mediated delivery of oncolytic viruses. Biochim Biophys Acta (2008) 1785:217–31 10.1016/j.bbcan.2008.02.001 18328829 PMC2888475

[4] Breitbach CJ Paterson JM Lemay CG Falls TJ McGuire A Parato KA Targeted inflammation during oncolytic virus therapy severely compromises tumor blood flow. Mol Ther (2007) 15:1686–93 10.1038/sj.mt.6300215 17579581

[5] Liu TC Hwang T Park BH Bell J Kirn DH The targeted oncolytic poxvirus JX-594 demonstrates antitumoral, antivascular, and anti-HBV activities in patients with hepatocellular carcinoma. Mol Ther (2008) 16:1637–42 10.1038/mt.2008.143 18628758

[6] Breitbach CJ Arulanandam R De Silva N Thorne SH Patt R Daneshmand M Oncolytic vaccinia virus disrupts tumor-associated vasculature in humans. Cancer Res (2013) 73:1265–75 10.1158/0008-5472.CAN-12-2687 23393196

[7] Diaz RM Galivo F Kottke T Wongthida P Qiao J Thompson J Oncolytic immunovirotherapy for melanoma using vesicular stomatitis virus. Cancer Res (2007) 67:2840–8 10.1158/0008-5472.CAN-06-3974 17363607

[8] Prestwich RJ Errington F Ilett EJ Morgan RS Scott KJ Kottke T Tumor infection by oncolytic reovirus primes adaptive antitumor immunity. Clin Cancer Res (2008) 14:7358–66 10.1158/1078-0432.CCR-08-0831 19010851 PMC2701231

[9] Prestwich RJ Ilett EJ Errington F Diaz RM Steele LP Kottke T Immune-mediated antitumor activity of reovirus is required for therapy and is independent of direct viral oncolysis and replication. Clin Cancer Res (2009) 15:4374–81 10.1158/1078-0432.CCR-09-0334 19509134 PMC4821072

[10] Wongthida P Diaz RM Galivo F Kottke T Thompson J Pulido J Type III IFN interleukin-28 mediates the antitumor efficacy of oncolytic virus VSV in immune-competent mouse models of cancer. Cancer Res (2010) 70:4539–49 10.1158/0008-5472.CAN-09-4658 20484025 PMC3896099

[11] Sobol PT Boudreau JE Stephenson K Wan Y Lichty BD Mossman KL Adaptive antiviral immunity is a determinant of the therapeutic success of oncolytic virotherapy. Mol Ther (2011) 19:335–44 10.1038/mt.2010.264 21119618 PMC3034857

[12] Diaconu I Cerullo V Hirvinen ML Escutenaire S Ugolini M Pesonen SK Immune response is an important aspect of the antitumor effect produced by a CD40L-encoding oncolytic adenovirus. Cancer Res (2012) 72:2327–38 10.1158/0008-5472.CAN-11-2975 22396493

[13] Huang PY Guo JH Hwang LH Oncolytic Sindbis virus targets tumors defective in the interferon response and induces significant bystander antitumor immunity in vivo. Mol Ther (2012) 20:298–305 10.1038/mt.2011.245 22068428 PMC3277240

[14] Workenhe ST Simmons G Pol JG Lichty BD Halford WP Mossman KL Immunogenic HSV-mediated oncolysis shapes the antitumor immune response and contributes to therapeutic efficacy. Mol Ther (2014) 22:123–31 10.1038/mt.2013.238 24343053 PMC3978812

[15] Matzinger P The danger model: a renewed sense of self. Science (2002) 296:301–5 10.1126/science.1071059 11951032

[16] Medzhitov R Janeway CA Jr Decoding the patterns of self and nonself by the innate immune system. Science (2002) 296:298–300 10.1126/science.1068883 11951031

[17] Tang D Kang R Coyne CB Zeh HJ Lotze MT PAMPs and DAMPs: signal 0s that spur autophagy and immunity. Immunol Rev (2012) 249:158–75 10.1111/j.1600-065X.2012.01146.x 22889221 PMC3662247

[18] Zou W Immunosuppressive networks in the tumour environment and their therapeutic relevance. Nat Rev Cancer (2005) 5:263–74 10.1038/nrc1586 15776005

[19] Naik S Russell SJ Engineering oncolytic viruses to exploit tumor specific defects in innate immune signaling pathways. Expert Opin Biol Ther (2009) 9:1163–76 10.1517/14712590903170653 19637971

[20] Kim M Williamson CT Prudhomme J Bebb DG Riabowol K Lee PW The viral tropism of two distinct oncolytic viruses, reovirus and myxoma virus, is modulated by cellular tumor suppressor gene status. Oncogene (2010) 29:3990–6 10.1038/onc.2010.137 20473328 PMC4374435

[21] Young AM Archibald KM Tookman LA Pool A Dudek K Jones C Failure of translation of human adenovirus mRNA in murine cancer cells can be partially overcome by L4-100K expression in vitro and in vivo. Mol Ther (2012) 20:1676–88 10.1038/mt.2012.116 22735379 PMC3437579

[22] Janeway C Immunogenicity signals 1,2,3 … and 0. Immunol Today (1989) 10:283–6 10.1016/0167-5699(89)90081-9 2590379

[23] Janeway CA Jr Approaching the asymptote? Evolution and revolution in immunology. Cold Spring Harb Symp Quant Biol (1989) 54(Pt 1):1–13 10.1101/SQB.1989.054.01.003 2700931

[24] Janeway CA Jr Medzhitov R Innate immune recognition. Annu Rev Immunol (2002) 20:197–216 10.1146/annurev.immunol.20.083001.084359 11861602

[25] Bianchi ME DAMPs, PAMPs and alarmins: all we need to know about danger. J Leukoc Biol (2007) 81:1–5 10.1189/jlb.0306164 17032697

[26] Tapia K Kim WK Sun Y Mercado-López X Dunay E Wise M Defective viral genomes arising in vivo provide critical danger signals for the triggering of lung antiviral immunity. PLoS Pathog (2013) 9:e1003703 10.1371/journal.ppat.1003703 24204261 PMC3814336

[27] Takeuchi O Akira S Pattern recognition receptors and inflammation. Cell (2010) 140:805–20 10.1016/j.cell.2010.01.022 20303872

[28] Joffre O Nolte MA Sporri R Reis e Sousa C Inflammatory signals in dendritic cell activation and the induction of adaptive immunity. Immunol Rev (2009) 227:234–47 10.1111/j.1600-065X.2008.00718.x 19120488

[29] Zanoni I Granucci F Regulation of antigen uptake, migration, and lifespan of dendritic cell by toll-like receptors. J Mol Med (Berl) (2010) 88:873–80 10.1007/s00109-010-0638-x 20556351

[30] Matzinger P Tolerance, danger, and the extended family. Annu Rev Immunol (1994) 12:991–1045 10.1146/annurev.immunol.12.1.991 8011301

[31] Kono H Rock KL How dying cells alert the immune system to danger. Nat Rev Immunol (2008) 8:279–89 10.1038/nri2215 18340345 PMC2763408

[32] Shi Y Evans JE Rock KL Molecular identification of a danger signal that alerts the immune system to dying cells. Nature (2003) 425:516–21 10.1038/nature01991 14520412

[33] Scheibner KA Lutz MA Boodoo S Fenton MJ Powell JD Horton MR Hyaluronan fragments act as an endogenous danger signal by engaging TLR2. J Immunol (2006) 177:1272–81 16818787 10.4049/jimmunol.177.2.1272

[34] Klune JR Dhupar R Cardinal J Billiar TR Tsung A HMGB1: endogenous danger signaling. Mol Med (2008) 14:476–84 10.2119/2008-00034.Klune 18431461 PMC2323334

[35] Guo ZS Liu Z Bartlett DL Tang D Lotze MT Life after death: targeting high mobility group box 1 in emergent cancer therapies. Am J Cancer Res (2013) 3:1–20 23359863 PMC3555201

[36] Krysko DV Agostinis P Krysko O Garg AD Bachert C Lambrecht BN Emerging role of damage-associated molecular patterns derived from mitochondria in inflammation. Trends Immunol (2011) 32:157–64 10.1016/j.it.2011.01.005 21334975

[37] Krysko DV Garg AD Kaczmarek A Krysko O Agostinis P Vandenabeele P Immunogenic cell death and DAMPs in cancer therapy. Nat Rev Cancer (2012) 12:860–75 10.1038/nrc3380 23151605

[38] Casares N Pequignot MO Tesniere A Ghiringhelli F Roux S Chaput N Caspase-dependent immunogenicity of doxorubicin-induced tumor cell death. J Exp Med (2005) 202:1691–701 10.1084/jem.20050915 16365148 PMC2212968

[39] Lindenmann J Klein PA Viral oncolysis: increased immunogenicity of host cell antigen associated with influenza virus. J Exp Med (1967) 126:93–108 10.1084/jem.126.1.93 4290961 PMC2138306

[40] Melcher A Todryk S Hardwick N Ford M Jacobson M Vile RG Tumor immunogenicity is determined by the mechanism of cell death via induction of heat shock protein expression. Nat Med (1998) 4:581–7 10.1038/nm0598-581 9585232

[41] Obeid M Tesniere A Ghiringhelli F Fimia GM Apetoh L Perfettini JL Calreticulin exposure dictates the immunogenicity of cancer cell death. Nat Med (2007) 13:54–61 10.1038/nm1523 17187072

[42] Tesniere A Apetoh L Ghiringhelli F Joza N Panaretakis T Kepp O Immunogenic cancer cell death: a key-lock paradigm. Curr Opin Immunol (2008) 20:504–11 10.1016/j.coi.2008.05.007 18573340

[43] Green DR Ferguson T Zitvogel L Kroemer G Immunogenic and tolerogenic cell death. Nat Rev Immunol (2009) 9:353–63 10.1038/nri2545 19365408 PMC2818721

[44] Bartlett DL Liu Z Sathaiah M Ravindranathan R Guo Z He Y Oncolytic viruses as therapeutic cancer vaccines. Mol Cancer (2013) 12:103 10.1186/1476-4598-12-103 24020520 PMC3847443

[45] Inoue H Tani K Multimodal immunogenic cancer cell death as a consequence of anticancer cytotoxic treatments. Cell Death Differ (2014) 21:39–49 10.1038/cdd.2013.84 23832118 PMC3857623

[46] Tesniere A Panaretakis T Kepp O Apetoh L Ghiringhelli F Zitvogel L Molecular characteristics of immunogenic cancer cell death. Cell Death Differ (2008) 15:3–12 10.1038/sj.cdd.4402269 18007663

[47] Lamkanfi M Dixit VM Manipulation of host cell death pathways during microbial infections. Cell Host Microbe (2010) 8:44–54 10.1016/j.chom.2010.06.007 20638641

[48] Ito H Aoki H Kühnel F Kondo Y Kubicka S Wirth T Autophagic cell death of malignant glioma cells induced by a conditionally replicating adenovirus. J Natl Cancer Inst (2006) 98:625–36 10.1093/jnci/djj161 16670388

[49] Alonso MM Jiang H Yokoyama T Xu J Bekele NB Lang FF Delta-24-RGD in combination with RAD001 induces enhanced anti-glioma effect via autophagic cell death. Mol Ther (2008) 16:487–93 10.1038/sj.mt.6300400 18253154

[50] Baird SK Aerts JL Eddaoudi A Lockley M Lemoine NR McNeish IA Oncolytic adenoviral mutants induce a novel mode of programmed cell death in ovarian cancer. Oncogene (2008) 27:3081–90 10.1038/sj.onc.1210977 18071311 PMC2493060

[51] Rodriguez-Rocha H Gomez-Gutierrez JG Garcia-Garcia A Rao XM Chen L McMasters KM Adenoviruses induce autophagy to promote virus replication and oncolysis. Virology (2011) 416:9–15 10.1016/j.virol.2011.04.017 21575980 PMC3113480

[52] Miyamoto S Inoue H Nakamura T Yamada M Sakamoto C Urata Y Coxsackievirus B3 is an oncolytic virus with immunostimulatory properties that is active against lung adenocarcinoma. Cancer Res (2012) 72:2609–21 10.1158/0008-5472.CAN-11-3185 22461509

[53] Donnelly OG Errington-Mais F Steele L Hadac E Jennings V Scott K Measles virus causes immunogenic cell death in human melanoma. Gene Ther (2013) 20:7–15 10.1038/gt.2011.205 22170342 PMC3378495

[54] Guo ZS Naik A O’Malley ME Popovic P Demarco R Hu Y The enhanced tumor selectivity of an oncolytic vaccinia lacking the host range and antiapoptosis genes SPI-1 and SPI-2. Cancer Res (2005) 65:9991–8 10.1158/0008-5472.CAN-05-1630 16267024

[55] Huang B Sikorski R Kirn DH Thorne SH Synergistic anti-tumor effects between oncolytic vaccinia virus and paclitaxel are mediated by the IFN response and HMGB1. Gene Ther (2011) 18:164–72 10.1038/gt.2010.121 20739958

[56] John LB Howland LJ Flynn JK West AC Devaud C Duong CP Oncolytic virus and anti-4-1BB combination therapy elicits strong anti-tumor immunity against established cancer. Cancer Res (2012) 72:1651–60 10.1158/0008-5472.CAN-11-2788 22315352

[57] Whilding LM Archibald KM Kulbe H Balkwill FR Öberg D McNeish IA Vaccinia virus induces programmed necrosis in ovarian cancer cells. Mol Ther (2013) 21:2074–86 10.1038/mt.2013.195 23985697 PMC3831043

[58] Borde C Barnay-Verdier S Gaillard C Hocini H Maréchal V Gozlan J Stepwise release of biologically active HMGB1 during HSV-2 infection. PLoS One (2011) 6:e16145 10.1371/journal.pone.0016145 21283827 PMC3023802

[59] Angelova AL Grekova SP Heller A Kuhlmann O Soyka E Giese T Complementary induction of immunogenic cell death by oncolytic parvovirus H-1PV and gemcitabine in pancreatic cancer. J Virol (2014). 10.1128/JVI.03688-13 24574398 PMC4019131

[60] Liikanen I Ahtiainen L Hirvinen ML Bramante S Cerullo V Nokisalmi P Oncolytic adenovirus with temozolomide induces autophagy and antitumor immune responses in cancer patients. Mol Ther (2013) 21:1212–23 10.1038/mt.2013.51 23546299 PMC3681222

[61] Endo Y Sakai R Ouchi M Onimatsu H Hioki M Kagawa S Virus-mediated oncolysis induces danger signal and stimulates cytotoxic T-lymphocyte activity via proteasome activator upregulation. Oncogene (2008) 27:2375–81 10.1038/sj.onc.1210884 17982491

[62] Colunga AG Laing JM Aurelian L The HSV-2 mutant DeltaPK induces melanoma oncolysis through nonredundant death programs and associated with autophagy and pyroptosis proteins. Gene Ther (2010) 17:315–27 10.1038/gt.2009.126 19798049 PMC2904070

[63] Wang Q Imamura R Motani K Kushiyama H Nagata S Suda T Pyroptotic cells externalize eat-me and release find-me signals and are efficiently engulfed by macrophages. Int Immunol (2013) 25:363–72 10.1093/intimm/dxs161 23446850

[64] Ito N DeMarco RA Mailliard RB Han J Rabinowich H Kalinski P Cytolytic cells induce HMGB1 release from melanoma cell lines. J Leukoc Biol (2007) 81:75–83 10.1189/jlb.0306169 16968820

[65] Moehler M Zeidler M Schede J Rommelaere J Galle PR Cornelis JJ Oncolytic parvovirus H1 induces release of heat-shock protein HSP72 in susceptible human tumor cells but may not affect primary immune cells. Cancer Gene Ther (2003) 10:477–80 10.1038/sj.cgt.7700591 12768193

[66] Grekova S Aprahamian M Giese N Schmitt S Giese T Falk CS Immune cells participate in the oncosuppressive activity of parvovirus H-1PV and are activated as a result of their abortive infection with this agent. Cancer Biol Ther (2010) 10:1280–9 10.4161/cbt.10.12.13455 21124075

[67] Grekova SP Raykov Z Zawatzky R Rommelaere J Koch U Activation of a glioma-specific immune response by oncolytic parvovirus minute virus of mice infection. Cancer Gene Ther (2012) 19:468–75 10.1038/cgt.2012.20 22539062

[68] Radic M Marion T Monestier M Nucleosomes are exposed at the cell surface in apoptosis. J Immunol (2004) 172:6692–700 15153485 10.4049/jimmunol.172.11.6692

[69] Uratsuji H Tada Y Kawashima T Kamata M Hau CS Asano Y P2Y6 receptor signaling pathway mediates inflammatory responses induced by monosodium urate crystals. J Immunol (2012) 188:436–44 10.4049/jimmunol.1003746 22102722

[70] Elankumaran S Rockemann D Samal SK Newcastle disease virus exerts oncolysis by both intrinsic and extrinsic caspase-dependent pathways of cell death. J Virol (2006) 80:7522–34 10.1128/JVI.00241-06 16840332 PMC1563725

[71] Meng C Zhou Z Jiang K Yu S Jia L Wu Y Newcastle disease virus triggers autophagy in U251 glioma cells to enhance virus replication. Arch Virol (2012) 157:1011–8 10.1007/s00705-012-1270-6 22398914 PMC7087167

[72] Washburn B Schirrmacher V Human tumor cell infection by Newcastle disease virus leads to upregulation of HLA and cell adhesion molecules and to induction of interferons, chemokines and finally apoptosis. Int J Oncol (2002) 21:85–93 10.3892/ijo.21.1.85 12063554

[73] Fournier P Arnold A Wilden H Schirrmacher V Newcastle disease virus induces pro-inflammatory conditions and type I interferon for counter-acting Treg activity. Int J Oncol (2012) 40:840–50 10.3892/ijo.2011.1265 22102168

[74] Zamarin D Holmgaard RB Subudhi SK Park JS Mansour M Palese P Localized oncolytic virotherapy overcomes systemic tumor resistance to immune checkpoint blockade immunotherapy. Sci Transl Med (2014) 6:226–32 10.1126/scitranslmed.3008095 24598590 PMC4106918

[75] Errington F Steele L Prestwich R Harrington KJ Pandha HS Vidal L Reovirus activates human dendritic cells to promote innate antitumor immunity. J Immunol (2008) 180:6018–26 18424722 10.4049/jimmunol.180.9.6018

[76] Fournier P Schirrmacher V Oncolytic Newcastle disease virus as cutting edge between tumor and host. Biology (2013) 2:936–75 10.3390/biology2030936 PMC3960873 24833054

[77] Clarke P Meintzer SM Gibson S Widmann C Garrington TP Johnson GL Reovirus-induced apoptosis is mediated by TRAIL. J Virol (2000) 74:8135–9 10.1128/JVI.74.17.8135-8139.2000 10933724 PMC112347

[78] Berger AK Danthi P Reovirus activates a caspase-independent cell death pathway. MBio (2013) 4:e178–113 10.1128/mBio.00178-13 23674612 PMC3656442

[79] Chi PI Huang WR Lai IH Cheng CY Liu HJ The p17 nonstructural protein of avian reovirus triggers autophagy enhancing virus replication via activation of phosphatase and tensin deleted on chromosome 10 (PTEN) and AMP-activated protein kinase (AMPK), as well as dsRNA-dependent protein kinase (PKR)/eIF2alpha signaling pathways. J Biol Chem (2012) 288:3571–84 10.1074/jbc.M112.390245 23233667 PMC3561576

[80] Meng S Jiang K Zhang X Zhang M Zhou Z Hu M Avian reovirus triggers autophagy in primary chicken fibroblast cells and Vero cells to promote virus production. Arch Virol (2012) 157:661–8 10.1007/s00705-012-1226-x 22241622

[81] Thirukkumaran CM Shi ZQ Luider J Kopciuk K Gao H Bahlis N Reovirus modulates autophagy during oncolysis of multiple myeloma. Autophagy (2013) 9:413–4 10.4161/auto.22867 23322106 PMC3590261

[82] Errington F White CL Twigger KR Rose A Scott K Steele L Inflammatory tumour cell killing by oncolytic reovirus for the treatment of melanoma. Gene Ther (2008) 15:1257–70 10.1038/gt.2008.58 18401435 PMC4821075

[83] Levine B Deretic V Unveiling the roles of autophagy in innate and adaptive immunity. Nat Rev Immunol (2007) 7:767–77 10.1038/nri2161 17767194 PMC7097190

[84] Chakrabarti A Ghosh PK Banerjee S Gaughan C Silverman RH RNase L triggers autophagy in response to viral infections. J Virol (2012) 86:11311–21 10.1128/JVI.00270-12 22875977 PMC3457150

[85] English L Chemali M Duron J Rondeau C Laplante A Gingras D Autophagy enhances the presentation of endogenous viral antigens on MHC class I molecules during HSV-1 infection. Nat Immunol (2009) 10:480–7 10.1038/ni.1720 19305394 PMC3885169

[86] Alexander DE Ward SL Mizushima N Levine B Leib DA Analysis of the role of autophagy in replication of herpes simplex virus in cell culture. J Virol (2007) 81:12128–34 10.1128/JVI.01356-07 17855538 PMC2169004

[87] Comber JD Robinson TM Siciliano NA Snook AE Eisenlohr LC Functional macroautophagy induction by influenza A virus without a contribution to major histocompatibility complex class II-restricted presentation. J Virol (2010) 85:6453–63 10.1128/JVI.02122-10 21525345 PMC3126538

[88] Michaud M Martins I Sukkurwala AQ Adjemian S Ma Y Pellegatti P Autophagy-dependent anticancer immune responses induced by chemotherapeutic agents in mice. Science (2011) 334:1573–7 10.1126/science.1208347 22174255

[89] Ayna G Krysko DV Kaczmarek A Petrovski G Vandenabeele P Fésüs L ATP release from dying autophagic cells and their phagocytosis are crucial for inflammasome activation in macrophages. PLoS One (2012) 7:e40069 10.1371/journal.pone.0040069 22768222 PMC3386917

[90] Thorburn J Horita H Redzic J Hansen K Frankel AE Thorburn A Autophagy regulates selective HMGB1 release in tumor cells that are destined to die. Cell Death Differ (2009) 16:175–83 10.1038/cdd.2008.143 18846108 PMC2605182

[91] Dengjel J Schoor O Fischer R Reich M Kraus M Müller M Autophagy promotes MHC class II presentation of peptides from intracellular source proteins. Proc Natl Acad Sci U S A (2005) 102:7922–7 10.1073/pnas.0501190102 15894616 PMC1142372

[92] Li Y Wang LX Yang G Hao F Urba WJ Hu HM Efficient cross-presentation depends on autophagy in tumor cells. Cancer Res (2008) 68:6889–95 10.1158/0008-5472.CAN-08-0161 18757401 PMC2905686

[93] Wei J Waithman J Lata R Mifsud NA Cebon J Kay T Influenza A infection enhances cross-priming of CD8+ T cells to cell-associated antigens in a TLR7- and type I IFN-dependent fashion. J Immunol (2010) 185:6013–22 10.4049/jimmunol.1002129 20956347

[94] Gauvrit A Brandler S Sapede-Peroz C Boisgerault N Tangy F Gregoire M Measles virus induces oncolysis of mesothelioma cells and allows dendritic cells to cross-prime tumor-specific CD8 response. Cancer Res (2008) 68:4882–92 10.1158/0008-5472.CAN-07-6265 18559536

[95] Uhl M Kepp O Jusforgues-Saklani H Vicencio JM Kroemer G Albert ML Autophagy within the antigen donor cell facilitates efficient antigen cross-priming of virus-specific CD8+ T cells. Cell Death Differ (2009) 16:991–1005 10.1038/cdd.2009.8 19229247

[96] Li Y Wang LX Pang P Cui Z Aung S Haley D Tumor-derived autophagosome vaccine: mechanism of cross-presentation and therapeutic efficacy. Clin Cancer Res (2011) 17:7047–57 10.1158/1078-0432.CCR-11-0951 22068657 PMC3495614

[97] Meng S Xu J Wu Y Ding C Targeting autophagy to enhance oncolytic virus-based cancer therapy. Expert Opin Biol Ther (2013) 13:863–73 10.1517/14712598.2013.774365 23488666

[98] Lowe SW Ruley HE Stabilization of the p53 tumor suppressor is induced by adenovirus 5 E1A and accompanies apoptosis. Genes Dev (1993) 7:535–45 10.1101/gad.7.4.535 8384579

[99] Debbas M White E Wild-type p53 mediates apoptosis by E1A, which is inhibited by E1B. Genes Dev (1993) 7:546–54 10.1101/gad.7.4.546 8384580

[100] Perez D White E TNF-alpha signals apoptosis through a bid-dependent conformational change in Bax that is inhibited by E1B 19K. Mol Cell (2000) 6:53–63 10.1016/S1097-2765(00)00007-1 10949027

[101] White E Sabbatini P Debbas M Wold WS Kusher DI Gooding LR The 19-kilodalton adenovirus E1B transforming protein inhibits programmed cell death and prevents cytolysis by tumor necrosis factor alpha. Mol Cell Biol (1992) 12:2570–80 1317006 10.1128/mcb.12.6.2570-2580.1992 PMC364450

[102] Marcellus RC Teodoro JG Charbonneau R Shore GC Branton PE Expression of p53 in Saos-2 osteosarcoma cells induces apoptosis which can be inhibited by Bcl-2 or the adenovirus E1B-55 kDa protein. Cell Growth Differ (1996) 7:1643–50 8959332

[103] Benedict CA Norris PS Prigozy TI Bodmer JL Mahr JA Garnett CT Three adenovirus E3 proteins cooperate to evade apoptosis by tumor necrosis factor-related apoptosis-inducing ligand receptor-1 and -2. J Biol Chem (2001) 276:3270–8 10.1074/jbc.M008218200 11050095

[104] Orvedahl A Alexander D Tallóczy Z Sun Q Wei Y Zhang W HSV-1 ICP34.5 confers neurovirulence by targeting the beclin 1 autophagy protein. Cell Host Microbe (2007) 1:23–35 10.1016/j.chom.2006.12.001 18005679

[105] Cassady KA Gross M Roizman B The second-site mutation in the herpes simplex virus recombinants lacking the gamma134.5 genes precludes shutoff of protein synthesis by blocking the phosphorylation of eIF-2alpha. J Virol (1998) 72:7005–11 9696792 10.1128/jvi.72.9.7005-7011.1998 PMC109920

[106] Randazzo BP Tal-Singer R Zabolotny JM Kesari S Fraser NW Herpes simplex virus 1716, an ICP 34.5 null mutant, is unable to replicate in CV-1 cells due to a translational block that can be overcome by coinfection with SV40. J Gen Virol (1997) 78(Pt 12):3333–9 9400985 10.1099/0022-1317-78-12-3333

[107] Chou J Roizman B The gamma 1(34.5) gene of herpes simplex virus 1 precludes neuroblastoma cells from triggering total shutoff of protein synthesis characteristic of programed cell death in neuronal cells. Proc Natl Acad Sci U S A (1992) 89:3266–70 10.1073/pnas.89.8.3266 1314384 PMC48847

[108] Jerome KR Fox R Chen Z Sears AE Lee Hy Corey L Herpes simplex virus inhibits apoptosis through the action of two genes, Us5 and Us3. J Virol (1999) 73:8950–7 10516000 10.1128/jvi.73.11.8950-8957.1999 PMC112926

[109] Zhou J Sun XY Fernando GJ Frazer IH The vaccinia virus K2L gene encodes a serine protease inhibitor which inhibits cell-cell fusion. Virology (1992) 189:678–86 10.1016/0042-6822(92)90591-C 1641985

[110] Dobbelstein M Shenk T Protection against apoptosis by the vaccinia virus SPI-2 (B13R) gene product. J Virol (1996) 70:6479–85 8709286 10.1128/jvi.70.9.6479-6485.1996 PMC190684

[111] Wasilenko ST Banadyga L Bond D Barry M The vaccinia virus F1L protein interacts with the proapoptotic protein Bak and inhibits Bak activation. J Virol (2005) 79:14031–43 10.1128/JVI.79.22.14031-14043.2005 16254338 PMC1280199

[112] Cooray S Bahar MW Abrescia NG McVey CE Bartlett NW Chen RA Functional and structural studies of the vaccinia virus virulence factor N1 reveal a Bcl-2-like anti-apoptotic protein. J Gen Virol (2007) 88:1656–66 10.1099/vir.0.82772-0 17485524 PMC2885619

[113] Everett H Barry M Lee SF Sun X Graham K Stone J M11L: a novel mitochondria-localized protein of myxoma virus that blocks apoptosis of infected leukocytes. J Exp Med (2000) 191:1487–98 10.1084/jem.191.9.1487 10790424 PMC2213443

[114] Macen JL Graham KA Lee SF Schreiber M Boshkov LK McFadden G Expression of the myxoma virus tumor necrosis factor receptor homologue and M11L genes is required to prevent virus-induced apoptosis in infected rabbit T lymphocytes. Virology (1996) 218:232–7 10.1006/viro.1996.0183 8615027

[115] Upton JW Kaiser WJ Mocarski ES Virus inhibition of RIP3-dependent necrosis. Cell Host Microbe (2010) 7:302–13 10.1016/j.chom.2010.03.006 20413098 PMC4279434

[116] Gannagé M Dormann D Albrecht R Dengjel J Torossi T Rämer PC Matrix protein 2 of influenza A virus blocks autophagosome fusion with lysosomes. Cell Host Microbe (2009) 6:367–80 10.1016/j.chom.2009.09.005 19837376 PMC2774833

[117] Zhirnov OP Klenk HD Influenza A virus proteins NS1 and hemagglutinin along with M2 are involved in stimulation of autophagy in infected cells. J Virol (2013) 87:13107–14 10.1128/JVI.02148-13 24027311 PMC3838240

[118] Yi C Liu X Liu Y Lu S Qi Y Hemagglutinin protein of measles virus induces apoptosis of HeLa cells via both extrinsic and intrinsic pathways. Can J Microbiol (2013) 59:814–24 10.1139/cjm-2013-0544 24313454

[119] Meiffren G Joubert PE Grégoire IP Codogno P Rabourdin-Combe C Faure M Pathogen recognition by the cell surface receptor CD46 induces autophagy. Autophagy (2010) 6:299–300 10.4161/auto.6.2.11132 20087059

[120] Park MS García-Sastre A Cros JF Basler CF Palese P Newcastle disease virus V protein is a determinant of host range restriction. J Virol (2003) 77:9522–32 10.1128/JVI.77.17.9522-9532.2003 12915566 PMC187425

[121] Igney FH Krammer PH Death and anti-death: tumour resistance to apoptosis. Nat Rev Cancer (2002) 2:277–88 10.1038/nrc776 12001989

[122] Liu P Xu B Shen W Zhu H Wu W Fu Y Dysregulation of TNFalpha-induced necroptotic signaling in chronic lymphocytic leukemia: suppression of CYLD gene by LEF1. Leukemia (2012) 26:1293–300 10.1038/leu.2011.357 22157808

[123] Alameda JP Moreno-Maldonado R Navarro M Bravo A Ramírez A Page A An inactivating CYLD mutation promotes skin tumor progression by conferring enhanced proliferative, survival and angiogenic properties to epidermal cancer cells. Oncogene (2010) 29:6522–32 10.1038/onc.2010.378 20838385

[124] Han W Li L Qiu S Lu Q Pan Q Gu Y Shikonin circumvents cancer drug resistance by induction of a necroptotic death. Mol Cancer Ther (2007) 6:1641–9 10.1158/1535-7163.MCT-06-0511 17513612

[125] Kaufman HL Bines SD OPTIM trial: a phase III trial of an oncolytic herpes virus encoding GM-CSF for unresectable stage III or IV melanoma. Future Oncol (2010) 6:941–9 10.2217/fon.10.66 20528232

[126] Liu TC Hallden G Wang Y Brooks G Francis J Lemoine N An E1B-19 kDa gene deletion mutant adenovirus demonstrates tumor necrosis factor-enhanced cancer selectivity and enhanced oncolytic potency. Mol Ther (2004) 9:786–803 10.1016/j.ymthe.2004.03.017 15194046

[127] Workenhe ST Pol JG Lichty BD Cummings DT Mossman KL Combining oncolytic HSV-1 with immunogenic cell death-inducing drug mitoxantrone breaks cancer immune tolerance and improves therapeutic efficacy. Cancer Immunol Res (2013) 1:309–19 10.1158/2326-6066.CIR-13-0059-T 24777969

[128] Cheng PH Lian S Zhao R Rao XM McMasters KM Zhou HS Combination of autophagy inducer rapamycin and oncolytic adenovirus improves antitumor effect in cancer cells. Virol J (2013) 10:293 10.1186/1743-422X-10-293 24059864 PMC3850263

[129] Heo J Reid T Ruo L Breitbach CJ Rose S Bloomston M Randomized dose-finding clinical trial of oncolytic immunotherapeutic vaccinia JX-594 in liver cancer. Nat Med (2013) 19:329–36 10.1038/nm.3089 23396206 PMC4268543

[130] Kim HS Kim-Schulze S Kim DW Kaufman HL Host lymphodepletion enhances the therapeutic activity of an oncolytic vaccinia virus expressing 4-1BB ligand. Cancer Res (2009) 69:8516–25 10.1158/0008-5472.CAN-09-2522 19843856

[131] Li J O’Malley M Urban J Sampath P Guo ZS Kalinski P Chemokine expression from oncolytic vaccinia virus enhances vaccine therapies of cancer. Mol Ther (2011) 19:650–7 10.1038/mt.2010.312 21266959 PMC3070102

[132] Yu F Wang X Guo ZS Bartlett DL Gottschalk SM Song XT T-cell engager-armed oncolytic vaccinia virus significantly enhances antitumor therapy. Mol Ther (2014) 22:102–11 10.1038/mt.2013.240 24135899 PMC3978793

[133] Hu W Davis JJ Zhu H Dong F Guo W Ang J Redirecting adaptive immunity against foreign antigens to tumors for cancer therapy. Cancer Biol Ther (2007) 6:1773–9 10.4161/cbt.6.11.4855 17986853 PMC2387205

[134] Kanerva A Nokisalmi P Diaconu I Koski A Cerullo V Liikanen I Antiviral and antitumor T-cell immunity in patients treated with GM-CSF-coding oncolytic adenovirus. Clin Cancer Res (2013) 19:2734–44 10.1158/1078-0432.CCR-12-2546 23493351

[135] Workenhe ST Mossman KL Oncolytic virotherapy and immunogenic cancer cell death: sharpening the sword for improved cancer treatment strategies. Mol Ther (2014) 22:251–6 10.1038/mt.2013.220 24048442 PMC3916036

[136] Janelle V Langlois MP Lapierre P Charpentier T Poliquin L Lamarre A The strength of the T cell response against a surrogate tumor antigen induced by oncolytic VSV therapy does not correlate with tumor control. Mol Ther (2014). 10.1038/mt.2014.34 24590047 PMC4048893

